# Do diurnal changes in blood pressure affect myocardial work indices?

**DOI:** 10.1111/jch.14377

**Published:** 2021-10-26

**Authors:** Cesare Cuspidi, Stefano Carugo, Marijana Tadic

**Affiliations:** ^1^ Department of Medicine and Surgery University of Milano‐Bicocca Milano Italy; ^2^ Department of Clinical Sciences and Community Health University of Milano and Fondazione Ospedale Maggiore IRCCS Policlinico di Milano Milano Italy; ^3^ University Clinical Hospital Centre “Dragisa Misovic” Belgrade Serbia

In this issue of the Journal, Li and coworkers^1^ report the findings of a study aimed at assessing the influence of brachial blood pressure (BP) changes over the course of a single day on myocardial work (MW) indices in normotensive and hypertensive individuals. Before commenting the results and clinical implications of the study, as well as its strengths and limitations, some more general considerations on current evidence on this research area and related issues can be useful to offer a comprehensive view of the topic.

Conventional echocardiographic parameters such as left ventricular (LV) end‐diastolic/end‐systolic volume and LV ejection fraction (LVEF) are considered reliable indicators to identify the outcome and risk of cardiovascular events in a wide range of clinical settings.^2^, ^3^ Despite the fact that LVEF derived from 2D calculation according to the modified Simpson method has long been considered as the most sensitive parameter of LV systolic function with high prognostic value, it has a number of inherent limitations that significantly reduce its capacity to show real LV performance. LVEF expresses endocardial fiber shortening, not focusing on mid‐wall myocardial fibers that are primarily responsible for LVEF, much more than subendocardial fibres.^4^ Furthermore, dependence on hemodynamic load, limited reproducibility and suboptimal inter/intra‐observer variability can alter the accuracy of the assessment of systolic function. Finally, subclinical LV systolic dysfunction usually cannot be unmasked by LVEF.^5^

The recently developed 2D and 3D speckle tracking echocardiography (a technique based on the analysis of interference patterns and acoustic reflections of myocardial motion and deformation) represents a valuable tool for the detection of subtle systolic dysfunction (which is of particular clinical interest in the hypertensive setting), and allows estimation of myocardial strain components. The advantage of LV strain is the comprehensive evaluation of multidirectional LV functions (longitudinal, circumferential, and radial), corresponding to the activation of myocardial layers (endocardial, mid‐myocardial, and epicardial) and to the different motions (longitudinal stretching, circumferential myocardial contraction, radial thickening).

Among parameters of LV mechanics, global longitudinal strain (GLS) represents a highly sensitive index of systolic function with a greater predictive power for cardiovascular outcomes than LVEF in a large spectrum of cardiovascular diseases including systemic hypertension.^6^ Furthermore, GLS has been proven to be a more reproducible parameter than LVEF and significantly less affected by load conditions.^7^

The role of myocardial strain and GLS as a new, more sensitive marker of cardiac organ damage in hypertension is currently supported by many individual studies. A recent meta‐analysis by our group including 4276 individuals (2089 normotensive controls and 2187 hypertensive patients) from 22 studies showed that GLS was significantly reduced in hypertensive patients compared to normotensive controls and this was the case even when the confounding effect of age was removed by comparing age‐matched patients, whereas systolic function measured by conventional LVEF was similar between groups.^8^ Notably, compared to controls, hypertensive patients exhibited increased LV mass, more concentric geometry, and impaired diastolic function.

Despite these promising evidence, the role of load conditions (ie, increased after‐load) remains particularly relevant in the hypertensive heart disease and could be an important confounder in the association between GLS and outcome. It has been suggested that increased after‐load can alter per se GLS and generate false conclusions about myocardial contractility. In this regard a meta‐analysis by Yingchoncharoen and coworkers^9^ based on 24 studies, who had enrolled a total of 2597 healthy patients, showed that normal values of GLS varied from −15.9% to −22.1%, highlighting a significant heterogeneity and inconsistency between studies. The meta‐regression carried out to investigate the causes of variability documented that BP, but not age, sex, frame rate, or equipment, was associated with variation in normal GLS values.

Russel and coworkers reported for the first time that MW, which considers both GLS and afterload, might overcome load‐dependence as one of limitations associated with GLS evaluation.^10^ They demonstrated that LV pressure‐strain loop area, which reflects regional LV MW, can be clinically measured by non‐invasive method that combines non‐invasively estimated LV pressure curve (ie, peripheral systolic BP) with strain obtained by speckle tracking echocardiography. MW measures LV work performed in systole and during isovolumic relaxation and represents a novel set of parameters of myocardial function. In the last decade numerous studies have evaluated the clinical role of the non‐invasively assessed MW and emphasized its high feasibility, limited observer variability, diagnostic sensitivity, and prognostic value makes it a valuable tool for clinical and research purposes. MW has been shown to be a more sensitive marker of systolic dysfunction than LVEF and GLS in patients with heart failure, coronary artery disease, valvular heart disease, hypertensive heart disease, cardiac dyssynchrony, hypertrophic cardiomyopathy and amyloidosis.^11^ The incremental value of MW as a prognostic marker for survival and hospitalizations has been also reported, especially in patients with chronic heart failure.^12^

As for hypertension, only a few studies have provided information on subclinical cardiac damage assessed by MW indices so far. A study by Jaglan and coworkers^13^ carried‐out in 65 stage 1 and two hypertensive patients and 15 controls showed that GLS and LVEF were preserved between the groups with no significant difference, whereas there was a statically significant difference in global MW index, global constructive MW and global wasted MW between controls and hypertensive patients. An association between impaired MW indices and LV hypertrophy (LVH) has been demonstrated among 105 untreated essential hypertensive patients compared to 55 normal controls.^14^ Global wasted MW was significantly increased in hypertensive patients with LVH compared with their counterparts without LVH and normotensive controls, while global MW efficiency was significantly reduced. Notably, ROC analysis revealed that combined global MW values were a more sensitive predictor of LV subclinical dysfunction than GLS. The largest study available to date comprising a total of 204 participants (45 controls, 70 patients with well controlled hypertension, 58 with uncontrolled hypertension, and 31 with resistant hypertension) documented that MW was significantly deteriorated in patients with uncontrolled and resistant hypertension compared to well‐controlled hypertensive patients and controls.^15^

In a study targeting MW in secondary hypertension, including 50 patients with primary aldosteronism, 50 age‐ and sex‐matched patients with essential hypertension, and 25 normotensive control individuals, Chen et al.^16^ found that global MW efficiency was lowest in primary aldosteronism, intermediate in essential hypertension, highest in normotensive controls and the opposite trend was evident for the global wasted MW.

Starting from the premise that hypertension represents a condition in which LV systolic function is strongly influenced by the after load and that BP in the hypertensive setting shows large fluctuations even over the course of a single day, Li and coworkers^1^ investigated, for the first time, the effect of daily BP changes on MW indices. For this purpose they simultaneously performed standard and 2D speckle tracking echocardiography, in accordance with current international guidelines, and measured brachial BP with a validated electronic device in a total of 117 participants (34 normotensive controls and 83 uncomplicated, untreated hypertensive patients). As for the demographic and clinical characteristics of hypertensive patients, the mean age of the was 56 years, with a similar prevalence of the two sexes, 11% of them were diabetic, 54% had grade 1 hypertension, and the overall prevalence of LVH was 40%. The acquisition of the data of interest was carried out twice in the same day by the same operator at 9:00 a.m. and at 5:00 p.m.. The calculation of daily BP changes was performed by comparing the morning and afternoon systolic BP values and considering the lowest value, regardless of the time sequence, as the baseline.

The magnitude of systolic BP changes during the study day were approximately 5 mm Hg in normotensive participants, 17 mm Hg in grade 1 hypertensive and 24 mm Hg in grade 2 hypertensive patients. Systolic BP variations in the normotensive group were not associated to significant changes of either the GLS or the MW indices (with the only exception of global constructive MW). On the contrary, in the hypertensive group the echocardiographic session study performed in the presence of the highest systolic values of the day showed a significant deterioration of GLS, as well as MW indices (ie, increased global MW index, global constructive MW, global wasted MW). In multivariable analysis changes in systolic BP were more closely related to changes in LV systolic function as assessed by GLS and MW, clearly pointing out the importance of systolic BP as a key marker of after load and a primary therapeutic target. More importantly, the results of this study contribute to strengthening the view on the pivotal role of BP variability in the genesis of organ damage.^17^

In conclusions, the study by Li and coworkers^1^ represents an important further step in the understanding the complex mechanisms underlying LV systolic function, highlighting, in particular, the role of short‐term systolic BP variations on cardiac mechanics. Although the findings by the present study must be considered with some caution (in relation to the limits correctly acknowledged by the authors, that is, small study sample from a single center), they actually open new perspectives in the evaluation of hypertensive mediated organ damage. In fact, the identification of systolic dysfunction defined by deterioration of LV mechanics in patients with high BP levels should be confirmed or excluded by repeating an echocardiographic examination when BP is adequately controlled in order to rule out the confounding effect of increased after‐load. In the new clinical scenario, the limitation that the analysis of cardiac mechanics is time consuming and demand special expertise may be overcome in the near future by the implementation of fully automated measurement of GLS and other parameters of LV mechanics that will use artificial intelligence methods.^18^

## CONFLICTS OF INTEREST

The authors report no conflicts of interest.
